# Nanosensor-based imaging of realtime dopamine release in neurons derived from iPSCs of patients with Parkinson's disease

**DOI:** 10.1016/j.mtbio.2025.101485

**Published:** 2025-01-19

**Authors:** Nayeon Lee, Dakyeon Lee, Jae Hyeok Lee, Bo Seok Lee, Sungjee Kim, Jae Ho Kim, Sanghwa Jeong

**Affiliations:** a Convergence Stem Cell Research Center, Medical Research Institute, Pusan National University, Yangsan, 50612, Gyeongsangnam-do, Republic of Korea; b Department of Physiology, Pusan National University School of Medicine, Yangsan, 50612, Gyeongsangnam-do, Republic of Korea; c School of Biomedical Convergence Engineering, Pusan National University, Yangsan, 50612, Gyeongsangnam-do, Republic of Korea; d Department of Chemistry, Pohang University of Science and Technology, Pohang, 37673, Republic of Korea; e Department of Neurology, School of Medicine, Pusan National University Yangsan Hospital, Yangsan, 50612, Gyeongsangnam-do, Republic of Korea

**Keywords:** Near-infrared fluorescence, Dopamine release, Carbon nanotube, Real-time visualization, Parkinson's disease

## Abstract

Dopamine (DA) is an essential neuromodulator that underlies critical aspects of cognitive processes, motor function, and reward systems. Disruptions in DA signaling contribute to various neurodegenerative diseases, including Parkinson's disease (PD). Despite its important role in neuronal function, the impact of DA release/uptake on neurochemical imbalances during neuronal development remains unclear. We propose a novel application of near-infrared catecholamine nanosensor (NIRCat) for real-time visualization of DA neurotransmission among neurodegenerative disease cells. The near-infrared fluorescence (900–1400 nm) of NIRCat allows the semi-quantitative measurement of DA release in living neurons and offers insights into cellular dynamics and neuropathological development. In this study, we applied NIRCat to elucidate DA release in human induced pluripotent stem cells (hiPSCs)-derived dopaminergic neurons from both healthy control and PD patient carrying GBA1 mutations. We accurately quantified electrically stimulated DA release events, identifying distinct ‘hotspots’ of activity across DA neuronal cells. Our findings present a significantly enhanced spatial and temporal resolution of DA signaling, providing a deeper understanding of the role and balance of DA release in the progression of neurodegenerative disease.

## Graphical abstract

**Figure:**
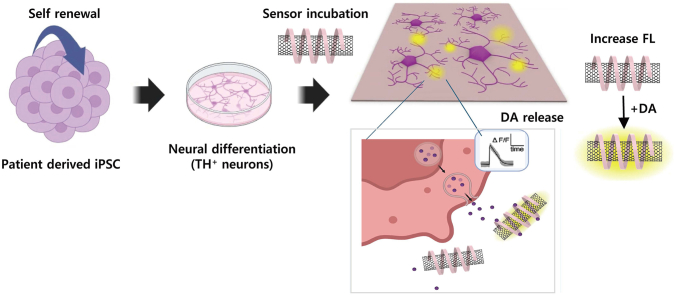

## 1 Introduction

An important genetic risk factor linked to Parkinson's disease (PD) is the mutations in the *GBA1* gene which encodes the lysosomal enzyme glucocerebrosidase (GCase), and it has recently been the most genetic abnormality [1,2]. *GBA1* mutations commonly cause both Gaucher disease and PD, significantly elevating the risk of developing PD by about 20-30-fold. Despite these findings, the precise pathogenic mechanism underlying *GBA1* mutation-associated PD (*GBA1*-PD) remains unclear. Kim et al. [3] reported that impaired GCase function can accelerate α-synuclein (α-syn) accumulation and lead to Parkinsonism-like symptoms in animal models. A meta-analysis also demonstrated that the *GBA1* risk variant increases the likelihood of cognitive impairment by approximately threefold among patients with PD [4]. Furthermore, dopaminergic neurons from human-induced pluripotent stem cells (hiPSCs) of PD patients with *N409S* mutation were identified with increased α-syn, elevated endoplasmic reticulum stress, and lysosomal dysfunction [5]. Given that *GBA1*-PD is similar to sporadic PD but with an earlier age of onset and a more rapid cognitive and motor dysfunction, the dopaminergic neurons derived from *GBA1*-PD hiPSCs are highly useful models for measuring spatiotemporal DA release in neurons of patients with PD.

Visualizing intracellular DA efflux is crucial for understanding DA signaling dynamics in neurons. The development of real-time DA measurement techniques in iPSC-derived neurons is essential, as it enables precise monitoring of DA release and distribution. These techniques are particularly important for the study of neurodegenerative and neuropsychiatric diseases, where it is critical to elucidate the mechanisms underlying these disorders and improve therapeutic approaches. To determine the DA levels in neuronal cells, various techniques, including positron emission tomography (PET), fast-scan cyclic voltammetry (FSCV) [6], microdialysis methods combined with high-performance liquid chromatograph (HPLC) [7], and other electrochemical methodologies [8] have been developed. To measure neural DA secretion, DA releases must be examined at the subcellular level with a spatial resolution that discriminates individual varicosities and with a temporal resolution of millisecond synaptic activities. However, common electrochemical DA detection methods cannot characterize the spatial dynamics of large numbers of synapses and dendritic-release events due to their low spatial resolution.

To leverage the detection of DA release with high spatiotemporal resolution simultaneously, carbon-based materials have been applied as promising candidates for sensitive and interference-free DA detection using near-infrared (NIR) fluorescence [9,10]. Specifically, single-walled carbon nanotubes (SWCNTs), are a monolayer graphene rolled up like a tube, have shown remarkable potential for sensing applications owing to their exceptional mechanical, electrical, and optical properties [11,12]. The utilization of SWCNTs for real-time visualization of catecholamine neuromodulator, specifically DA, has been studied for understanding the neurotransmission of neuromodulators beyond the synaptic cleft and interaction with extra-synaptic metabotropic receptors [9,10,13]. The SWCNT, specifically noncovalently functionalized with single strand (GT)_6_ oligonucleotides to form the NIR fluorescent catecholamine nanosensor, provide as a versatile platform for optical sensor which emits in the second near-infrared (NIR-II, 900–1700 nm) and recognizes molecules via their corona phase. NIR-II fluorescence mitigates the effect of tissue scattering within the emission window. This property has enabled through-cranium imaging, as NIR-II light penetrate biological tissue effectively compared to visible light [14]. SWCNT-based nanosenors exhibit robust non-photobleaching photostability, enabling their utilization in long-term imaging experiments. The nanosecond scale binding kinetics and nanoscale dimensions of SWCNT-based nanosenors improved sensitivity and specificity with temporal and spatial resolution in real time. Elizarova et al. utilized SWCNT sensors to detect DA release from cocultured neurons derived from mouse hippocampus and ventral mid-brain. This approach involved imaging the fluorescence emission resulting from the reversible binding of DA to the SWCNT sensors. Under electrical stimulation, they could observe the spatiotemporal DA release from large numbers of neuronal release sites simultaneously [10]. Bulumulla et al. visualized the spatiotemporal dynamics of DA release in dendritic processes and diffusion of DA with synaptic resolution using DopaFilm coated SWCNT sensors [9]. They applied optogenetic stimulus to evoke DA activity and examined the protein machinery involved in organizing release in dendritic processes. The SWCNT-based nanosensors could significantly advance understanding of neural signaling dynamics and facilitate the development of new diagnostic and therapeutic tools for neurological disorders.

Understanding the mechanisms of DA release/uptake during neuronal development is crucial to elucidating the causes of neurodegenerative disease. In the study, we used SWCNT-based nanosensors for real-time visualization of DA signaling in hiPSC-derived dopaminergic neurons of a patient with *GBA1*-PD. This innovative approach enabled us to track of DA release events with a high spatial and temporal resolution, thereby predict the interaction between DA neurotransmission and neurodegenerative disease progression.

## 2 Materials and methods

### 2.1 Generation of hiPSCs from a patient with *GBA1*-PD

Peripheral blood mononuclear cells (PBMCs) were isolated from the blood of a PD patient with mutations in *GBA1* gene and was reprogrammed using a non-integrated Sendai virus (Cytotune 2.0 Sendai virus, A16517, Thermo Fisher Scientific). Transduced cells were plated on Matrigel-coated culture dish in Stem Pro-34 medium (10639011, Thermo Fisher Scientific), and hiPSC colonies with sharp edges were visible after 14–18 days. The iPSC colonies were manually selected approximately 3 weeks post-viral transduction and expanded every five days in StemFlex medium (A3349401, Thermo Fisher Scientific). This study was approved by the IRB Board of Pusan National University Yangsan Hospital, Republic of Korea (#05-2021-009) [15,16].

### 2.2 Neural differentiation to dopaminergic neurons

For the dopaminergic neuron induction, the control and *GBA1*-PD hiPSCs were differentiated into neural cells as follows as three steps: neuroectoderm, neural progenitor (early) and neurite outgrowth (late). Both iPSCs were plated on Matrigel (354277, Corning) at a density of 15,000 cells/cm^2^. The initial differentiation media conditions included DMEM media (LM001-05-500, Thermo Fisher Scientific) with 15 % knock-out serum replacement (KSR) (10828028, Thermo Fisher Scientific) from day 1–6, and N2 supplement (17502048, Thermo Fisher Scientific) containing L-glutamine (25030081, Thermo Fisher Scientific) and nonessential amino acid (NEAA) (11140050, Thermo Fisher Scientific) from day 6–10. For neural induction and patterning, we used small molecules associated with SMAD inhibitors and WNT activator. Dual SMAD inhibitors, 0.2 μM LDN193189 (04–0074, Stemgent) and 10 μM SB431542 (13031, Cayman) were added from day 1–10 and 1 to 8, respectively. From days 2–10, we added 100 ng/ml Sonic hedgehog (SHH) (100-45, Peprotech) and 100 ng/ml FGF8 (100-25, Peprotech). The WNT signaling activator, 1 μM CHIR99021 (S2924, Selleckchem) was included from days 4–10. For the neural progenitor and neurite outgrowth, which included dopaminergic neurons from day 12, the media was added with N2 supplement, 20 ng/ml BDNF (450-02, Peprotech), 20 ng/ml GDNF (450-10, Peprotech), 500 μM dbcAMP (D0627, Sigma Aldrich) and 200 μM ascorbic acid (AA) (A4403, Sigma Aldrich). On day 15, cells were dissociated to single cells using Accutase (A1110501, Thermo Fisher Scientific) and plated on Matrigel-coated dishes.

### 2.3 Immunochemistry

hiPSCs and dopaminergic neurons were washed with phosphate-buffered saline (PBS) (LB001-01, Wellgene), fixed with 4 % formaldehyde (47608, Sigma Aldrich) in PBS for 10 min, and permeabilized using 0.1 % Triton X-100 (93443, Sigma Aldrich) in PBS. Cells were incubated for 1 h in a blocking solution (0.1 % Triton X-100 and 5 % BSA in PBS) at room temperature. Cells were incubated with primary antibodies in a blocking solution overnight. The primary antibodies used OCT4 (sc-5279, Santa Cruz), NANOG (ab109250, Abcam) and TRA1-81 (sc-21706, Santa Cruz) for hiPSC pluripotency, and SOX2 (23064S, Cell signaling), NESTIN (MAB5326, Millipore), TUJ1 (5568S, Cell signaling) and TH (58844S and 45648S, Cell signaling) for neural progenitor cells and dopaminergic neurons. Then, the cells were incubated with the appropriate secondary antibodies conjugated with fluorescence, Goat anti-Mouse IgG (H + L) conjugated with Alexa Fluor 568 (A1104, Thermo Fisher Scientific), Goat anti-rabbit IgG (H + L) conjugated with Alexa Fluor 488 (A11034, Thermo Fisher Scientific) with DAPI (D9542-5 MG, Sigma Aldrich) at room temperature for 1 h. Cell images were obtained using EVOS microscopy (EVOS 5000, Thermo Fisher Scientific). Data on specific cell populations were analyzed from microscopic images using ImageJ software.

### 2.4 Synthesis of NIRCat sensors

SWCNTs were purchased from NanoIntegris (raw small-diameter HiPco SWCNTs, batch #HS37-027) and 5′-GTG TGT GTG TGT-3′ single-stranded DNAs ((GT)_6_-ssDNAs) were purchased from Integrated DNA technology Inc. (standard desalting and lyophilized powder). (GT)_6_-ssDNAs were dispersed in DNase free water and diluted to a 1 mM stock solution in distilled water (DW). The NIRCat dispersion was fabricated by mixing 1 mg of HiPco SWCNT with 100 nmol of (GT)_6_ ssDNA in 1 ml of 1 × PBS. The mixture was dispersed through bath sonication for 5 min and probe tip sonication (SONICS VCX-130, tip diameter: 4 mm, 4 W) for 30 min in an ice-bath to form NIRCat via noncovalent adhesion of ssDNA on SWCNT surface. After sonication, (GT)_6_ ssDNA-SWCNT suspension was centrifuged at 21,000 g (Eppendorf 5425) for 1 h and the supernatant was collected and separated from the remaining unsuspended SWCNTs. The NIRCat nanosensors exhibit intrinsic NIR fluorescence emission ranging from 900 nm to 1500 nm depending on the chirality of the carbon lattice [17]. The nanosensor suspension was stored at 4 °C until further use and stored for no longer than one week.

### 2.5 Cell viability and toxicity

Neurons treated with NIRCat were assessed for cell viability using trypan blue dye. Neural cells were washed with PBS and detached from the dish by Accutase. A mixture was prepared as 10 μl of the cell suspension and an equal volume of trypan blue solution (H-T8154, Merck). Then, 10 μl of the mixture was transferred into a hemocytometer-counting chamber to count alive cells, are the results were presented as a percentage of cell viability relative to the control. To measure apoptotic cells, the control and *GBA1*-PD neurons cultured on a 24-well plate were washed with PBS, and 100 μl of propidium Iodide (S7112, Sigma Aldrich) was added before being incubated for 30 min in 37 °C in a 5 % CO_2_ incubator. The subsequent fluorescence was measured at a wavelength of 564 nm using a fluorescence microscope (EVOS 5000). Data on specific cell populations were determined from microscopic images using ImageJ software.

### 2.6 NIRCat characterization

For the optical characterization of NIRCat, its absorbance and fluorescence spectra of NIRCat were obtained and analyzed. The nanosensor solution concentration was determined by measuring the absorbance at 632 nm using the SWCNT extinction coefficient (0.036 mg L^−1^cm^−1^) [13]. The fluorescence was measured using an InGaAs photodiode array detector (NIRQuest, Ocean Optics) and fluorescence microscope (custom-built system based on Olympus IX73) excited with a 721-nm laser (CNI laser) was used for imaging the DA responses. The DA stock solution was prepared by dissolving DA hydrochloride (H8502, Sigma-Aldrich) in DW up to 10 mM. The fluorescence spectra of the NIRCat solution were measured at wavelengths of 900-1500 nm before and after the addition of DA. The DA dose-dependent response of NIRCat was determined by applying DA concentrations ranging from 1 to 10 mM in the solution phase and on a Matrigel coated cover glass. The ΔF/F_0_ values in solution phase were calculated from maximum peak at 1130 nm, mainly representing (9,4) chirality. For the experiment on the Matrigel-coated cover glass, the NIRCat nanosensors were coated over a layer of Matrigel and were calculated using the average ΔF/F_0_ value of each pixel in the field of view.

### 2.7 Fluorescence imaging setup for spatiotemporal DA monitoring

For NIR-II imaging, we developed a custom-built epifluorescence inverted microscope system based on an Olympus IX73. The system used two cameras to simultaneously image NIR-II and visible light. The visible light was excited using an LED light source and imaged using a scientific complementary metal oxide semiconductor (sCMOS) camera (CS2100M-USB, Thorlabs). NIR-II fluorescence was excited using a 721-nm laser and imaged using InGaAs camera (Ninox 640, Raptor Photonics). NIR-II imaging was performed at 5 frames/s for all live cell experiments.

### 2.8 Monitoring of DA release via electrical stimulation

The neuronal cultures on the coverslip were washed three times with 1 × PBS before NIRCat sensor treatment to avoid nonspeciﬁc adsorption and aggregation of SWCNTs by media and proteins. Then, 1 ml of 5 mg/L NIRCat nanosensor solution, including 10 mM D-(+)-glucose in 1 × PBS, was added to the coverslip and incubated for 10 min at 22 °C. The nanosensors were adsorbed onto the Matrigel around the neurons on the coverslip, and the unbound sensors were washed three times with 1 × PBS. Coverslips were mounted in an opened type imaging chamber with parallel electrodes (Chamlide-EC, Live Cell Instrument) on a 37 °C heating plate above the microscope for electrical stimulation during imaging. The chamber with imaging buffer was composed of 136 mM NaCl (81, Ducksan), 2.5 mM KCl (P5405, Sigma Aldrich), 2.0 mM CaCl_2_ (C7902, Sigma Aldrich), 1.3 mM MgCl_2_(M4880, Sigma Aldrich), 2 mM D-(+)-glucose (G7021, Sigma Aldrich), and 10 mM HEPES at pH 7.4 [10]. We used a 20 × objective lens to detect a dopaminergic neuron in the region within the parallel electrodes. We then switched to a 50 × objective lens to image the DA release in the dopaminergic neurons. Imaging was performed via electrical stimulation, generating a square-wave pulse at 20 Hz (5-s duration, 10V) from a stimulus generator (AFG-2225, GW Instek) through parallel electrodes for 5 s, during fluorescence imaging at 5 frames/s with a 200-ms exposure time. The fresh imaging buffer was perfused (2 ml/min, 37 °C) for 1 min before imaging another region on the coverslip. For testing the DA response of nanosensors, imaging was performed via the rapid addition of DA (final concentration of 100 μM) to the chamber followed by careful pipetting three times to facilitate faster diffusion.

### 2.9 Imaging processing and data analysis

Each imaging stack was processed using open-source MATLAB code (available at https://github.com/davidackerman/nnmf) that referenced a previously published material from the Beyene group [9]. The code computed the ΔF/F_0_ stack, where F_0_ was obtained by performing multiple rounds of low-pass filtering, and calculated temporal FWHM and amplitude of ΔF/F_0_ traces to find the location of hotspot as local maxima of the peak derivative. We overlayed the resulting regions of interest (ROIs) data and bright field images and analyzed ΔF/F_0_ at hotspot ROIs. The code detects DA release hotspots by analyzing regions of interest (ROIs) with a peak height variation of less than 500 %. However, this approach may incorrectly classify noise as a signal, resulting in false positives for the identification of DA release events. To address this, additional refinement steps were applied to the selected hotspot ROIs to manually exclude noise signals characterized by high frequencies, absent peaks, and low amplitudes. These steps minimized false positives and improved the reliability of hotspot detection (Fig. S1). We calculated the area of DA-release hotspots, where each pixel corresponds to 0.74 μm in length. To minimize the influence of impulse noise and reduce false-positive identifications, we applied a size threshold of 1.64 μm^2^, equivalent to the area of three combined pixels. This threshold ensures that only genuine fluorescence signals are included in the analysis, effectively distinguishing true hotspots from noise artifacts. From the resulting hotspots, evoked peak height and the average number of hotspots were calculated. The average numbers of hotspots were obtained by averaging the number of DA-evoked hotspots selected from each stimulus image stack to generate a slice average number of hotspots.

### 2.10 Statistical analysis

Statistical differences between groups were analyzed using the *t*-test and one-way analysis of variance (ANOVA) for multiple comparisons. The two-tailed Student's t-test was performed to compare the dopaminergic neurons. One-way ANOVA was performed using Tukey's post-hoc test for cell viability. The ΔF/F_0_ mean peak intensity, hotspot size, and hotspot number, were analyzed using an unpaired two-tailed *t*-test. All values are presented as mean ± standard error of the mean, and the *p*-values are indicated as follows: ∗*P* < 0.05, ∗∗*P* < 0.01, ∗∗∗*p* < 0.005 and ∗∗∗∗*p* < 0.001. All statistical analyses were performed using GraphPad Prism 9 (GraphPad Software, Inc.).

## 3 Results

### 3.1 Generation and neural differentiation of normal control and *GBA1* PD-hiPSCs

**Fig. 1:**
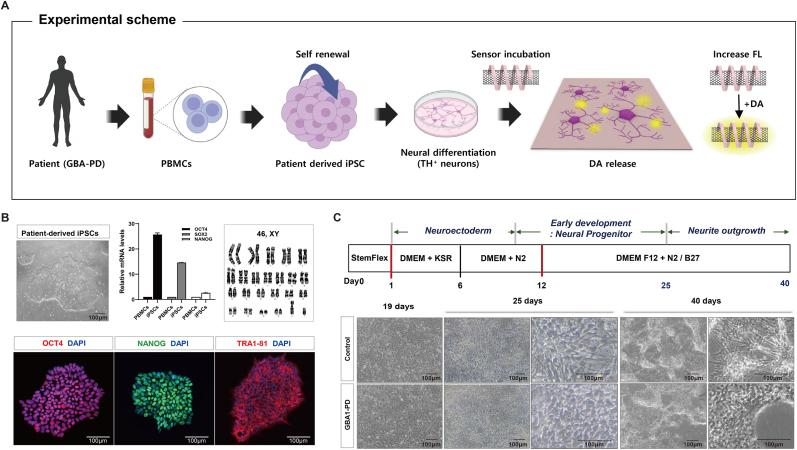
**hiPSC-based model for studying DA dynamics in Control and *GBA1*-PD.** (A) Schematic overview for the measurement of dopaminergic activity using an optical DA biosensor in hiPSC-derived neurons from Control and *GBA1*-PD patient. (B) Characterization of hiPSCs from a patient's PBMCs, detailing pluripotent stem cell markers. Scale bar: 100 μm. (C) Comparative neural differentiation yield between normal iPSCs (control) and *GBA1*-PD iPSCs over throughtout development, highlighting the cell morphologies at 19, 25 days and 40 days. Scale bar: 100 μm.

To establish an *in vitro* human cellular model for exploring DA release in normal control and *GBA1*-PD, we established hiPSC lines using general ‘Yamanaka's reprogramming method’ by transducing the PBMCs of patients with Sendai virus encoding OCT4, SOX2, KLF4 and c-MYC factors [18,19]. *GBA1*-PD patient-derived iPSC lines robustly expressed pluripotent markers, including OCT4, NANOG and TRA1-81, and consistently maintained a normal karyotype (Fig. 1A and B). As shown in Fig. 1C, hiPSCs were differentiated into neurons using a directed differentiation procedure that can reproducibly generate functional dopaminergic neurons, as previously reported by Song et al. [20]. We have efficiently differentiated neural cells that persist high levels across multiple batches of neurons through day 25, as indicated by the expression of differentiated progenitor cell markers SOX2, NESTIN and TUJ1 (Figs. S2A and S2B), as well as dopaminergic neuron markers NURR1 and TH (Figs. S2C and S2D). Both control and *GBA1*-PD hiPSC-derived NPCs were efficiently induced to produce mature neurons, including dopaminergic neurons. The initial stage of differentiation revealed no significant differences in SOX2 and NESTIN expression between the control and *GBA1*-PD neural cells. Conversely, mature neurons exhibited relatively lower MAP2 and TH expression in *GBA1*-PD neurons than in normal neurons (Fig. S2B). At day 40, the morphology of the GBA1-PD neurons in the late differentiation stage differed from that of normal neurons, reducing neurite neuron-to-neuron connections (Fig. 1C). It is possible that the early neural cells in GBA1-PD could have been delayed for the neurite outgrowth and connections because early neurogenesis is related to development and plasticity, which is impaired in chronic neurodegeneration, including PD [21,22].

### 3.2 Functional assessment of dopaminergic neurons derived from *GBA1* PD iPSCs

**Fig. 2:**
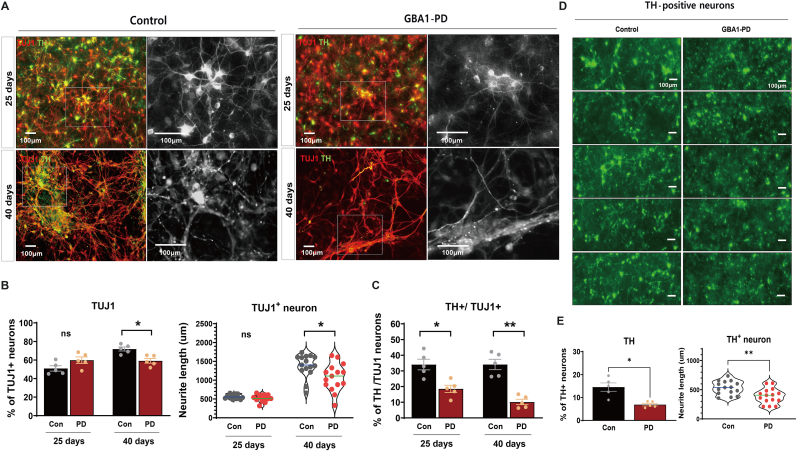
**Functional dopaminergic neurons derived from control and *GBA1*-PD iPSCs** (A) Neural cells from control and *GBA1*-PD iPSCs at 25 and 40 days, indicating maturation and synaptogenesis. Scale bar: 100 μm. (B) Quantitative representation of DA neurons among total neural cells. (C) Comparison of dopaminergic neurons in *GBA1*-PD neurons compared with control neurons at day 40. (D) Comparison of tyrosine hydroxylase (TH)-positive expressed neurons and neurite outgrowth between control and *GBA1*-PD neurons. Data are presented as mean ± SEM. Statistical significance was analyzed using a two-tailed Student's t-test, ∗*p* < 0.05, ∗∗*p* < 0.01.

To investigate the dopaminergic neurogenesis in *GBA1*-PD iPSC-derived neurons, we further differentiated the NPCs under mature differentiation conditions with neurotrophic factors, including BDNF, GDNF and dbcAMP. Neuronal maturation and functionality of dopaminergic neurons generated by NPCs and midbrain neurons are characterized by high expression of TH, a key enzyme in DA synthesis [[23], [24], [25]]. Moreover, an increased population of TH-positive cells within TUJ1-positive neurons, a neuron-specific marker, correlates with a higher portion of ability to develop into dopaminergic neurons [26,27]. Furthermore, during early differentiation (25 days), there was no significant difference in the percentage of neural cells and neurite length between the control and *GBA1*-PD neurons. However, by day 40, *GBA1*-PD neurons exhibited a lower population of TUJ1-positive neurons (Fig. 2A, 71.9 ± 4.8 % and 56.2 ± 5.7 %, for control and *GBA1*-PD neurons, respectively), similar to a reduction of neurite length in *GBA1*-PD neurons (Figs. 2B and 1385.1 ± 305 ㎛ and 1077.8 ± 363 ㎛ for control and *GBA1*-PD neurons, respectively). We also found that TH was expressed in dopaminergic neurons on the same days. Quantitative analysis showed that at day 40, the percentage of TH-positive cells among total neural cells was 34.0 ± 7.6 % and 18.5 ± 7.6 % in the control and *GBA1*-PD neurons, respectively. This implies the existence of mature dopaminergic neurons, which were significantly reduced in *GBA1*-PD neurons (Fig. 2C). Consistently, TH expression and neurite length were notably reduced in *GBA1*-PD neurons compared with the control (approximately 531 ± 116 ㎛ and 390.1 ± 130 ㎛, respectively (Fig. 2D and E), suggesting that *GBA1*-PD plays a role in the DA functional deficits during neural development. Moreover, patient-derived *GBA1* mutant dopaminergic neurons reportedly exhibit impaired cellular calcium homeostasis and DA synthesis in substantia nigra neurons via TH activation, the rate-limiting enzyme of catecholamine biosynthesis [28,29]. A previous study in *GBA1*-deficient mice showed significant loss in the stereological number of dopaminergic neurons in substantia nigra [30]. Overall, we focused on *GBA1*-PD hiPSCs displaying significant differences in neurite morphology and functionality when compared with the control neurons, especially at the late differentiation stage (40 days). *GBA1*-PD neurons also showed a decreased proportion of TH/TUJ1-positive neurons and shorter neurite lengths compared with the control neurons. These results suggest a consistent impairment in dopaminergic neuron development and maturation in *GBA1*-PD neurons. Therefore, it is crucial to focus on the disrupted functionality of dopaminergic neurons and to further determine whether DA release is similar to dopaminergic neurons dysfunction during neural development.

### 3.3 Synthesis and characterization of NIRCat nanosensor

**Fig. 3:**
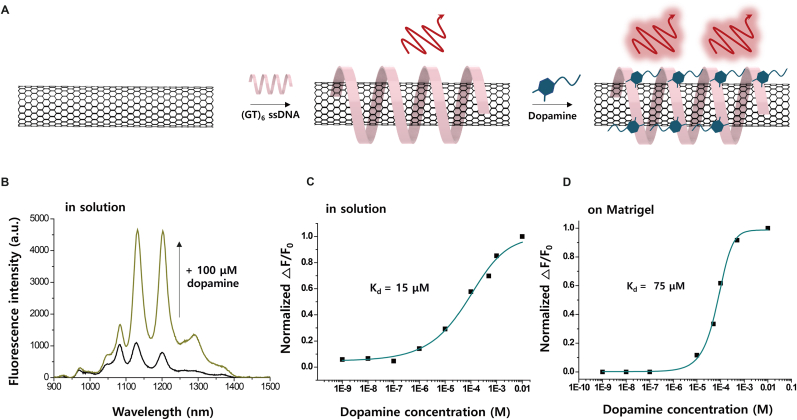
**Synthesis and calibration of NIRCat sensors.** (A) Schematic of the NIRCat synthesis. (B) Representative fluorescence response validating the sensitivity and specificity to DA. (C) Calibration curve and DA response kinetics of NIRCat in PBS solution (n = 3); three replicates each. (D) Dose-response curve on the Matrigel.

For the spatiotemporal imaging of DA release, we applied the (GT)_6_ ssDNA wrapped SWCNT nanosensor, NIRCat, which optically responds to DA concentration around the constructs [13]. The NIR-II ﬂuorescent SWCNT probe was functionalized with (GT)_6_ ssDNA, comprising repeated guanine and thymine sequences (Fig. S3). This functionalization resulted in nanosensors capable of fluorescence enhancement by recognizing DA, facilitating the imaging of synaptic and extrasynaptic catecholamines, along with their release and reuptake dynamics, in neuron cells (Fig. 3A). The sensing mechanism of NIRCat was suggested to involve DA interacting with the phosphate groups of DNA backbones via its hydroxy groups, thereby pulling the phosphate group towards the SWCNT surface [31]. This interaction suppresses quenching sites, thereby increasing the SWCNT fluorescence quantum yield [32]. Kruss et al. suggest a sensing mechanism, based on molecular dynamics (MD) simulations, in which DA interacts with the phosphate groups of the DNA backbone via its two hydroxyl groups [30,31]. This interaction brings the phosphate groups closer to the SWCNT surface, altering the local potential and removing quenching sites. As a result, the fluorescence quantum yield of the SWCNTs increases.

Krasley et al. propose an alternative mechanism, where the amine group of DA interacts with the negatively charged phosphate backbone of ssDNA-SWCNT, while the electron density on the catechol's aryl motif plays a crucial role in modulating fluorescence [33]. Using a combination of experimental approaches and MD simulations, they demonstrated that the optical modulation of DNA-SWCNT sensors is strongly influenced by the electrochemical properties of catechol. Specifically, DA stabilizes through π-stacking interactions with the SWCNT surface, while its protonated amine group forms electrostatic interactions with the ssDNA phosphate backbone. These interactions stabilize dopamine's binding and perturbs the local chemical environment of the nanotube, enhancing fluorescence. The fluorescence intensity of NIRCat sensors exhibited a significant increase of up to 560 % at a wavelength of 1200 nm following the addition of 100 μM DA (Fig. 3B). The distinct peaks in the fluorescence spectrum corresponded to different SWCNT chiralities within the samples. The DA-specific fluorescence response is rapidly reversible and changes with DA release and uptake [34]. The sensor response (ΔF/F_0_) was calculated using the peak of the (9,4) SWCNT chirality at 1130 nm to determine a calibration curve in PBS solution (Fig. 3C). The ΔF/F_0_ response of NIRCat was evaluated DA concentrations of 1 nM- 1 mM, ensuring dynamic responses to biologically relevant DA concentrations. The dissociation constant (K_d_) of the DA was obtained from the response curve at K_d_ = 15 μM. To assess the sensor response in the cultured cell environment, NIRCat was immobilized on a Matrigel-coated glass (Fig. 3D). The ΔF/F_0_ value of NIRCat was determined at DA concentrations similar to those under solution conditions. Physiological DA concentrations within synaptic vesicles could be very high (>100 mM) [35] but rapidly decline to the nM range within milliseconds after release [36]. To assess the specificity of the NIRCat probe, we measured its fluorescence response to various neurotransmitters, including DA, epinephrine (EP), γ-aminobutyric acid (GABA), glutamate (Glu), acetylcholine (ACh), and uric acid (UA). The normalized fluorescence changes (△F/F0) upon the addition of these analytes were quantified (Fig. S4). Our findings reveal that NIRCat is highly sensitive to DA and EP, while exhibiting negligible responses to GABA, Glu, ACh, and UA. The synthesis of EP requires the phenylethanolamine-N-methyltransferase (PNMT) enzyme, which is primarily expressed in the adrenal medulla and certain adrenergic neurons but is absent in dopaminergic neurons. Additionally, the experiments were conducted in a dopaminergic environment confirmed by TH expression in hiPSC-derived neurons, supporting the specificity of NIRCat for DA detection. The NIRCat nanosensors exhibited a robust turn-on optical response to DA, demonstrating a dynamic range suitable for in vivo neurophysiological applications.

### 3.4 Optimal conditions for exposure of the NIRCat nanosensor

**Fig. 4:**
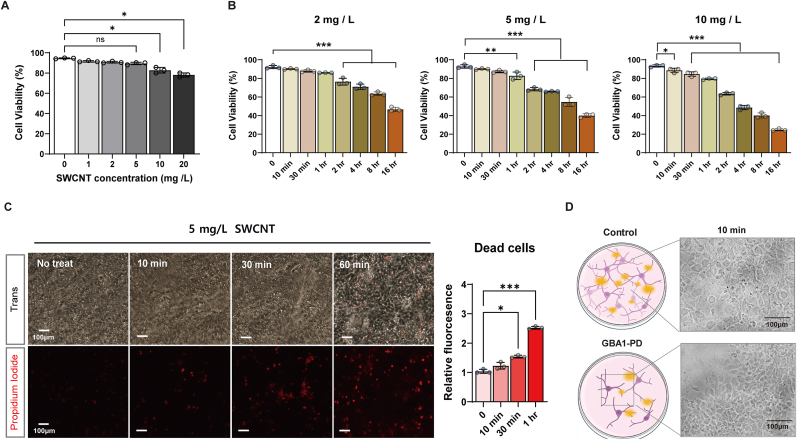
**Cytotoxic test depending on the different concentrations and times for NIRCat exposure in neuronal cells** (A) Cell viability of neural cells exposed to NIRCat at different concentrations of 1,2 5, 10 and 20 mg/L. (B) Cell viability of neural cells exposed to 2, 5 and 10 mg/L for time durations of 0–16 h. (C) Propidium Iodide (PI) staining for apoptotic cells exposed to 5 mg/L NIRCat. (D) Control and *GBA1*-PD neural cells after 5 mg/L NIRCat exposure for 10 min. Data are presented as mean ± SEM. Statistical significance for cell growth and percentages (%) of live cells were analyzed using ordinary one-way ANOVA, followed by Dunnett's multiple comparison's test: ns *p* < 0.05 ∗ *p* < 0.005. Statistical significance for cell growth and % of PI-stained cells were analyzed using ordinary one-way ANOVA, followed by Dunnett's multiple comparison's test: ∗*p* < 0.05, ∗∗*p* < 0.01, ∗∗∗*p* < 0.005.

**Fig. 5:**
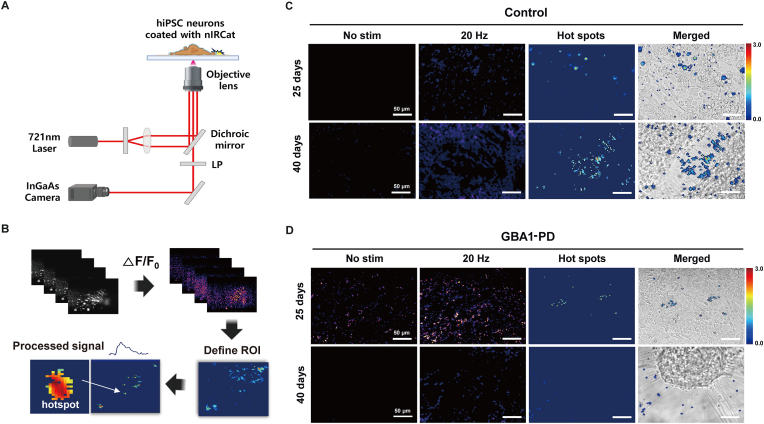
**Imaging fluorescence activity in both control and *GBA1*-PD neurons.** (A) Schematic depiction of the arrangement of optical components in a NIRCat imaging system. (B) Flow chart of DA release imaging processing in NIRCat fluorescence dynamics. Comparison dynamics of DA release between (C) control and (D) *GBA1*-PD neuronal cells on 25 and 40 days using NIRCat fluorescence modulation during a 20-Hz electrical stimulation for 5 s. The fluorescence hotspots occur upon DA release events in connection with neuron-to-neuron.

We evaluated the optimal conditions of nanosensor incubation, including incubation concentration and time for maximizing biocompatibility and sensor performance, using NIRCat exposure in normal hiPSC-derived neural cells. A viable condition for cells is important because it helps to assess the potentially harmful effect of NIRCat and ensure its safe use in biological systems on non-toxic SWCNT-based applications [37,38]. Cell viability was assessed using trypan blue assay to analyze the cytotoxic effect of ssDNA-SWCNT complexes on neural cells on day 25. The percentage of viable cells was found to be 94 ± 0.5 %, 91 ± 0.5 %, 90 ± 0.9 %, 00.89 ± 1.2 %, 82 ± 2.8 % and 78 ± 1.5 % at NIRCat concentrations of 0, 1, 2, 5, 10, and 20 mg/L, respectively, after incubating for 10 min (Fig. 4A). Cell viability appeared to be minimally affected by NIRCat concentration of ≤10 mg/L. To optimize the NIRCat exposure time of neural cells, we evaluated three different concentrations, 2, 5 and 10 mg/L depending on exposure time from 10 min to 16 h. As shown in Fig. 4, Fig. 2, Fig. 5 mg/L NIRCats significantly reduced the amount of viable cells ≥1 h, whereas 10 mg/L NIRCat reduced cell viability starting at 10 min. It is suggested that NIRCat concentrations of 2 or 5 mg/L with exposed for 1 h has comparatively low cytotoxicity compared with high concentrations of 10 or 20 mg/L. Based on the trypan blue assay, we selected NIRCat concentration of 5 mg/L with an incubation time of less than 30 min as being suitable for DA detection in neural cells and further investigated cell death in an incubation time-dependent manner. Previous studies have demonstrated that DNA-wrapped SWCNTs do not impact cell proliferation, with treated cells exhibiting similar growth rates to untreated cells at a concentration of 50 mg/L for 30 min [39,40]. However, in this similar system, DNA-wrapped SWCNTs have been shown to significantly affect hiPSC-derived neurons under similar conditions.

To investigate the apoptotic cell death, neural cells treated with 5 mg/L NIRCat were stained with propidium iodide (PI). PI staining is a fluorescent method that indicates loss of membrane integrity, where viable cells remain unstained and fluorescent PI binds to the DNA of dead cells. Therefore, the dead cells represented the red fluorescence signal. Beyond 30 min, dead cells were clearly detectable as time progressed (Fig. 4C). At 1 h, we observed a significant increase in the amount of apoptotic cells by approximately 7.7 ± 0.3 % compared with the untreated control (2.8 ± 0.4 %), which was more than 2.5-fold increase rather than control. It was also observed that beyond 8 h, the neurons changed phenotypically, and the number of apoptotic cells increased significantly (Figs. S5A and S5B), suggesting that the cytotoxicity of neural cells increased with the time of incubation beyond 30 min despite being treated with 5 mg/L NIRCat. Therefore, to measure the DA signaling from early to late neuronal differentiation, we treated both control and GBA1-PD neurons with 5 mg/L NIRCat for 10 min (Fig. 4D).

### 3.5 Imaging of dynamic DA release between normal neurons and *GBA1*-PD neurons

Imaging of dynamic DA release can be achieved using NIRCat, which offers high spatiotemporal resolution and emits NIR fluorescence [13]. Real-time visualization of dynamic DA release within control and *GBA1*-PD neurons was performed using an inverted microscope system equipped with an excitation wavelength of 721 nm and an InGaAs camera for detection (Fig. 5A). NIRCat sensors were mainly bound to both the Matrigel substrate and cellular membranes (Fig. S6). A 721-nm laser was used as the excitation light source in all imaging experiments. To observe the release of DA from the neurons, NIRCat sensors were treated with neuronal cells at a concentration of 5 mg/L and incubated for 10 min to coat the extracellular space. The cells were washed three times with PBS solution to remove unbound sensors. We used the bipolar electrodes in the imaging chamber, to generate electrical stimulation from a power supply and imaged before and after stimulation along the time. The stimulation triggered the release of dopaminergic neuronal axons, which facilitated DA diffusion upon its release. We performed NIRCat fluorescence imaging to visualize DA release and diffusion in control and *GBA1*-PD neurons before stimulation (No stim) and during a 20-Hz electrical stimulation for 5 s. As a positive control, 100 μM of DA was added to the sample at the end of each experiment to verify the nanosensor response to DA (Fig. S7). The relative change in fluorescence intensity (ΔF/F_0_) over time to compare the evoked fluorescence peaks of NIRCat before and after the electrical stimulation (Fig. 5B). The fluorescence hotspots correspond to the increases in local DA concentrations during DA release events. To identify DA release hotspots and calculate the maximal ΔF/F_0_ per hotspot, we processed the image stack using MATLAB code from a previous publication [9]. We obtained the DA release hotspots data, such as location and fluorescent intensity, and quantified their kinetics.

To assess the dynamic behavior of DA between the control and *GBA1*-PD neuronal cells, we imaged the cells at early (25 days) and late (40 days) stages of differentiation to capture the function and dysfunction of dopaminergic neurons during neural development (Fig. 5C and D). In normal control cells, a higher number of hotspots were observed during neuronal development at both early and late stages compared with *GBA1*-PD cells. In the early stage, NIRCat activation was widespread with increased fluorescence throughout the cell. At the late stage, normal neurons formed neurites with hotspots, indicating DA release and diffusion. *GBA1*-PD cells also formed late-stage neurites, but with different morphologies, exhibiting aggregation of neuron cells from control neurons, and DA release was observed near and on the neurites. A significant difference was observed in the number of hotspots that occurred in the disease model, suggesting altered DA dynamics in *GBA1*-PD neurons. In individual hotspots of *GBA1*-PD neurons, we also observed that NIRCat fluorescence changes in response to DA differed from control neurons (Fig. S8).

### 3.6 Comparison of dynamic DA release in the normal and *GBA1*-PD neurons

**Fig. 6:**
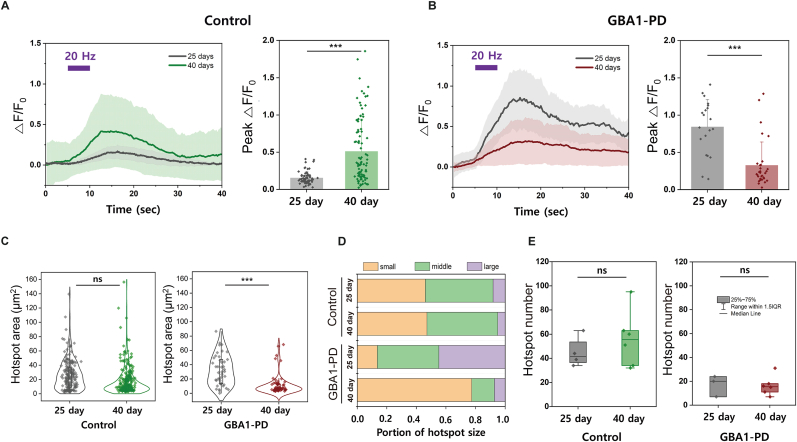
**Dynamic DA release patterns between control and *GBA1*-PD neurons.** Comparison of average NIRCat fluorescence change (ΔF/F_0_) of hotspots from (A) control and (B) *GBA1*-PD neural cells at 25 and 40 days. Time traces of ΔF/F_0_ following 20 Hz electrical stimulation for 5 s (left). Field-of-view (FOV) means traces of hotspots and SD bands are presented over n = 3 stimulation. The maximal peak ΔF/F_0_ of NIRCat after stimulation (right). Dots and bars represent each hotspot and the mean over n = 3 stimulation. (C) Distribution of hotspot size in control and *GBA1*-PD between 25 and 40 days. (D) The portion of hotspot size in control and *GBA1*-PD according to 25 and 40 days. Hotspot sizes were categorized as small (<3 μm), middle (3–6 μm), and large (>6 μm) and averaged for n = 3 stimulation. (E) Average hotspot number in control and *GBA1*-PD between 25 and 40 days. Dots and bars represent hotspot number in each FOV and the average of hotspot number in FOV. T-test was performed two-tailed Student's t-test, ∗*p* < 0.05, ∗∗*p* < 0.01, ∗∗∗*p* < 0.005.

To compare the DA release dynamics of *GBA1*-PD and control neural cells, we analyzed the DA release signals of the two groups upon electrical stimulation, which rapidly induces a local increase in DA levels around varicosities. *GBA1*-PD neurons exhibited significantly reduced neurite length and number compared with normal neurons (Fig. 2B). The NIRCats were thoroughly coated into the extracellular space to reflect DA diffusion away from release sites. The average peak ΔF/F_0_ for the entire hotspot indicates the relevant amount of DA released from neuron cells. Higher peak ΔF/F_0_ values suggest greater DA release, while lower peak ΔF/F_0_ values indicate reduced release. Following the 20-Hz electrical stimulation for 5 s, distinct DA responses to NIRCat (ΔF/F_0_) were observed in the control and *GBA1*-PD neurons. At the early stage (25 days), the average peak ΔF/F_0_ was 0.158 ± 0.084 in normal neurons, whereas significantly higher values of 0.845 ± 0.364 were measured in *GBA1*-PD neurons (Fig. 6A and B). At the late stage (40 days), the average peak ΔF/F_0_ of normal neuronal cells increased from 0.158 ± 0.084 to 0.513 ± 0.431, indicating an increase over maturation. In contrast, *GBA1*-PD neurons showed a decrease in the average peak ΔF/F_0_ from 0.845 ± 0.364 to 0.328 ± 0.312 over the same period.

The average hotspot size for normal neurons was not statistically different between early (26 ± 21 μm^2^) and late (170 ± 19 μm^2^) stages. However, the average hotspot size of GBA1-PD neurons was significantly reduced from 32 ± 21 μm^2^ to 11 ± 13 μm^2^. To investigate the size proportion of the hotspots, we categorized hotspot sizes as small (<9 μm^2^), middle (9–36 μm^2^), and large (>36 μm^2^) for 3 slices. GBA1-PD neurons in the late stage had no large hotspots (Fig. 6C). Contrarily, the size proportion of the control group hotspots showed negligible changes. These findings revealed the changing landscape of hotspot distribution and highlighted potential differences in DA secretion dynamics between the early and late differentiation stages of *GBA1*-PD neurons. Although the hotspot size in the *GBA1*-PD group decreased over time, no significant difference in the average hotspot number (per imaging area of 180 mm^2^) was observed between the early (17.0 ± 8.8) and late (16.3 ± 8.1) stages (Fig. 6E). The average hotspot number of the normal neurons differ marginally between the early (45.0 ± 12.6) and late (55.8 ± 23.1) stages. However, it is noteworthy that the hotspot number of *GBA1*-PD neurons was less than half that of the control group.

## 4 Discussions

In this study, we examined DA release in dopaminergic neurons derived from iPSCs of patients with PD and studied the difference in DA signaling between *GBA1*-PD and control neurons. This study proposes a novel approach to use SWCNT nanosensor for real-time visualization of DA neurotransmission within neurodegenerative disease cells. For the disease model, we generated iPSCs from *GBA1*-PD patient using Yamanaka reprogramming method [18,19,41], expressing pluripotent markers, and maintaining a normal karyotype. Normal and *GBA1*-PD hiPSCs were differentiated into neurons, including dopaminergic neurons, with successful generation of NPCs and mature neurons. *GBA1*-PD neurons differed phenotypically, including delayed neurite outgrowth and connections in comparison to normal neurons during late-stage differentiation (40 days).

Functional assessments of dopaminergic neurons revealed significant differences between the control and *GBA1*-PD. In previous studies, *GBA1*-mutant neurons altered cellular calcium homeostasis and DA synthesis in dopaminergic neurons in the substantia nigra, and the *GBA1*-PD animal model also showed significant loss of dopaminergic neurons [28,30]. Consistently, GBA1-PD neurons at 40 days showed a lower percentage of TUJ1-positive neurons, reduced neurite length, and decreased dopaminergic neurons that express TH. Therefore, *GBA1*-PD neurons exhibited impaired in both TH expression and neurite length compared to control neurons, suggesting potential deficits in DA function during neural development. In case of idiopathic PD, patients with *GBA1* mutations are characterized by an earlier age at onset, greater cognitive decline, and a greater likelihood of atypical clinical manifestations [42,43]. Thus, to compare the DA signals between normal and *GBA1*-PD neurons exposure to NIRCat may offer a good approach for a high-resolution and real-time visualization of DA signals, providing insights into DA release/uptake during neuropathological development. Its applications in comparing DA signals in neurodegenerative disease model, the use of NIRCat offers significant advantages due to its potential for rapid adaptation in detecting a various disease [9,44].

To measure the DA signals from live cells, we coated the intercellular Matrigel matrix with NIRCat nanosensors by co-incubation of NIRCat with cultured neural cells and optimized the NIRCat concentration and exposure time of on hiPSC-derived neural cells to minimize the harmful effect on neural cells. We also attempted to pre-coat the Matrigel substrate with NIRCat and differentiate the iPSCs into neural cells, as performed in a previous report [9], which used poly-lysine film as substrate and grew rat dopaminergic neuron (not iPSC). However, the hiPSCs on NIRCat pre-coated matrix were not successfully differentiated from the pristine matrix. It may be come from the complexity of neural differentiation from iPSCs and its sensitivity to the surrounding environment. To co-incubate the NIRCat with neural cells, it is necessary to understand the effect of NIRCat on these cells by which determines its cytotoxicity because SWCNTs may induce an increasing oxidation levels and dose-dependently decrease cell viability in brain tissue [37]. The trypan blue assay revealed the marginal cytotoxic effect of NIRCat nanosensors under 5 mg/L concentration and 30 min incubation. In the same condition, apoptotic cell death was also trivially observed using PI staining. Therefore, 10 min of incubation with 5 mg/L NIRCat were chosen as a suitable condition for monitoring DA release/uptake in hiPSC-derived neural cells. We found that hiPSC-derived neurons were more sensitive to the influence of NIRCat nanosensors compared with previous reports on cancer cells or fibroblast [[45], [46], [47]]. Nerve cells are more sensitive to oxidative stress and inflammation than other cell types, which may explain there are more sensitive to NIRCat [48,49].

*GBA1*-PD, a neuronal lineage derived from patient's PBMCs, differed markedly from normal neuronal cell line (control) in several crucial aspects: i) The propensity to differentiate into DA-releasing neurons decreases markedly during early developmental stages, with the number of DA-releasing hotspots reducing to ≤50 % of the normal density (Fig. 6D). ii) The spatial extent of these hotspots decreased progressively from early to late developmental stages. Initially, the average hotspot size was approximately 17 % larger than that of the control, but by the late stage, it was 19 % smaller than that of the control (Fig. 6C). iii) Assessment of the average peak ΔF/F_0_ within these hotspots revealed a substantial decline to 60 % of control levels in the late stage (Fig. 6B). This peak ΔF/F_0_ serves as a proxy for DA release and shows a striking 270 % increase in control from early to late stages, whereas *GBA1*-PD has a significant decline in comparison. This result indicates that DA dysfunction is progressive in *GBA1*-PD neurons, with a severe decline in DA release levels during later stages. In contrast, normal conditions exhibit increased DA secretion level in later stages, suggesting the potential for development of physiological functions. The marked contrast between the increasing DA levels in the control and the dramatic decrease in *GBA1*-PD neurons highlights the neuropathological phenomena associated with *GBA1*-PD.

Our study reveals that DA release and hotspot size in *GBA1*-PD neurons progressively decline over time, identifying impaired DA release as a hallmark of *GBA1*-PD. This aligns with the hypothesis by Cramb et al. that PD progression begins with reduced DA release, followed by axonal degeneration, neuronal loss, and downstream signaling disruptions [50]. Supporting this, PET imaging studies of individuals with familial PD risk show early presynaptic dopaminergic dysfunction, including reduced ^18^F-DOPA uptake, consistent with our observation of reduced DA release between 25 and 40 days in *GBA1*-PD neurons. Impaired DA transporter (DAT) function may underline these changes. α-Synuclein accumulation, driven by GBA1 mutations, has been shown to disrupt synaptic function before axonal degeneration. Lundblad et al. reported a 50 % reduction in DAT function in early PD [51]. We further confirmed the impaired DAT function by measuring DA clearance rates, finding a 3.5-fold increase in the decay time constant in GBA1-PD neurons compared to controls (Fig. S9). This slower reuptake rate highlights DAT dysfunction as a contributor to synaptic DA dysregulation. Overall, these results suggest that dopaminergic dysfunction in *GBA1*-PD neurons from both impaired reuptake and progressive synaptic impairments caused by α-synuclein accumulation, culminating in reduced DA release over time.

A study using PD mouse model showed that the early phenotype involves a decrease in synaptic activity, which progresses to motor dysfunction [52]. Moreover, a recent research, hiPSC-induced DA neurons from PD patients with E326K-GBA1 mutation has been found a reduction in sodium currents and a decrease in synaptic activity in DA neurons [53]. This release pattern in *GBA1*-PD neurons aligns with characteristics of neurodegenerative diseases such as PD, which leads to significant loss of dopaminergic neurons and decreased DA production. This finding supports the hypothesis that DA dysfunction plays a critical role in the neuropathological development of *GBA1*-PD. We provide insight into the evolving hotspot distribution landscape and highlight potential differences in DA secretion dynamics between the early and late stages of *GBA1*-PD neural development. Our nanosensor-based approach for extracellular DA dynamics detection will extend beyond PD to other neurodegenerative disorders, enabling real-time monitoring of neurotransmitter release in diverse model systems. This methodology could elucidate previously uncharacterized aspects of synaptic transmission and reveal novel mechanistic insights, particularly in the context of altered neurotransmitter homeostasis.

## 5 Conclusion

In this study, we present a significant advancement in our understanding of DA signaling in dopaminergic neurons derived from *GBA1*-PD patient-specific hiPSCs. By establishing a platform for the real-time visualization of DA neurotransmission using NIRCat nanosensor, we have enabled high-resolution monitoring of DA release within neurons affected by neurodegenerative diseases. We also easily detected DA dysfunction and compared DA release between *GBA1*-PD and normal neurons, highlighting the key differences in DA release patterns and phenotypical characteristics during neural development. These findings have significant translational potential, as dynamic DA signaling monitoring enables real-time assessment of both pharmacological and non-pharmacological therapeutic interventions in neurodegenerative disorders. Furthermore, our study offers valuable insights into neurodegenerative disease progression. The observed progressive decline in DA-releasing hotspots within GBA1-PD neurons illustrates the dynamic nature of DA signaling disruption over time.

To enhance experimental reliability and reduce bias, several key improvements would strengthen the study in the future: analyzing multiple timepoints during neuronal differentiation to capture developmental dynamics, diversifying the donor pool by using iPSC lines from both healthy controls and PD patients, and standardized preparation of nanosensors to minimize batch-to-batch variations. These modifications would ensure broader applicability of our findings while maintaining robust experimental control. Further noise reduction techniques, such as frequency-based and temporal filtering, statistical thresholding, and machine learning-based noise detection, can significantly enhance the accuracy and robustness of signal classification [54]. These approaches help isolate real signals from noise-related artifacts, improving the reliability and objectivity of data analysis. Future implementations of these methods will further refine our analytical workflow and ensure high-quality results.

By using single-chirality SWCNTs with distinct emission wavelengths, multiplexed imaging can be achieved to simultaneously detect multiple neurotransmitters based on their unique spectral properties. For example, NIRCat with (9,4) SWCNT emitting at 1100 nm could be applied with another (6,5) SWCNT nanosensor for serotonin [55], emitting at 995 nm, could be used for multiplexed neurotransmitter sensing. This provides a powerful tool for receptor-specific pharmacological studies in cell culture, enabling simultaneous monitoring of DA release and cell-type-specific neuronal activity, and real-time interactions between neurotransmitter systems. Our findings demonstrate the progressive nature of DA dysfunction in pathogenic PD, characterized by a marked decline in DA-releasing hotspots. Overall, our study identifies avenues for exploring potential therapeutic interventions targeting DA signaling in neurodegenerative disorders.

## CRediT authorship contribution statement

**Nayeon Lee:** Writing – review & editing, Writing – original draft, Methodology. **Dakyeon Lee:** Writing – review & editing, Writing – original draft, Methodology, Conceptualization. **Jae Hyeok Lee:** Methodology. **Bo Seok Lee:** Formal analysis. **Sungjee Kim:** Validation. **Jae Ho Kim:** Writing – review & editing, Writing – original draft, Supervision, Conceptualization. **Sanghwa Jeong:** Writing – review & editing, Supervision.

## Statement of significance

We explore an approach to real-time visualization of DA release in human induced pluripotent stem cells-derived dopaminergic neurons affected by GBA1 mutation associated PD. By utilizing near-infrared catecholamine nanosensors, we provide unprecedented insights into the spatiotemporal dynamics of neurotransmission. The findings uncover distinct DA release ‘hotspots’ and enhance the understanding of DA signaling role in neurodegeneration. This innovative measurement capability emphasizes the potential of SWCNT and NIRCat technologies in elucidating cellular dynamics and developing targeted therapies. Ultimately, our research represents a significant advance in biomaterials and neurobiology, bridging fundamental insights with clinical relevance for therapies targeting neurodegenerative disease.

## Declaration of competing interest

The authors declare that they have no known competing financial interests or personal relationships that could have appeared to influence the work reported in this paper.

## Data Availability

Data will be made available on request.
