# Supplementary material for: Nanosensor-based imaging of realtime dopamine release in neurons derived from iPSCs of patients with Parkinson's disease
Source: Mater Today Bio. 2025 Jan 19;31:101485. doi: 10.1016/j.mtbio.2025.101485 (PMC11791356; doi:10.1016/j.mtbio.2025.101485)
Supplement: Multimedia component 1 [file mmc1.docx]

**Supplemental information**

**Nanosensor-based Imaging of Realtime Dopamine Release in Neurons Derived from iPSCs of Patients with Parkinson’s Disease**

Nayeon Lee^1, 2, #^, Dakyeon Lee^3, 4, #^, Jae Hyeok Lee^5^, Bo Seok Lee^1, 2^, Sungjee Kim^4^,

Jae Ho Kim^1, 2, ⁋^ and Sanghwa Jeong^3, ⁋^

Supplementary Fig. 1. Signal classification and identification of dopamine-release hotspot

Supplementary Fig. 2. Neural differentiation of normal and *GBA1*-PD hiPSCs

Supplementary Fig. 3. Characterization of ssDNA-SWCNT and SC-SWCNT complexes

Supplementary Fig. 4. NIRCat optical response to 100 μM DA, EP, GABA, Glu, ACh, and 10 μM uric acid (UA) in PBS solution

Supplementary Fig. 5. PI staining for apoptotic cells exposed to 5 mg/L SWCNT from 2 h to 16 h

Supplementary Fig. 6. Brightfield and NIR fluorescence images of cultured cells incubated with NIRCat probes on a Matrigel substrate

Supplementary Fig. 7. NIRCat response to DA treatment

Supplementary Fig. 8. Comparison of DA release in individual hotspot between control and *GBA1*-PD

Supplementary Fig. 9. “Turn-off” rate of NIRCat nanosensors

**Supplemental information**

**Supplementary Fig. 1**


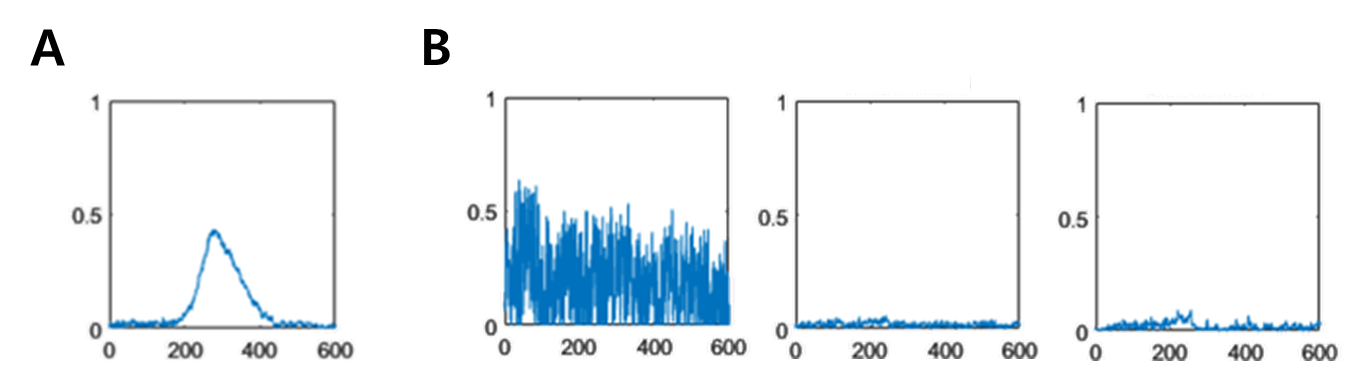


**Supplementary Fig. 1. Signal classification and identification of dopamine-release hotspot.** (A) Representative fluorescence data showing distinct and well-defined dopamine-release hotspots. (B) Examples of fluorescence signals classified as noise due to high frequency, lack of distinct signal peak, or significantly low amplitudes. These ambiguous signals, accounting for less than 10% of the data, were excluded from the analysis to ensure accuracy and reliability.

**Supplementary Fig. 2**

**
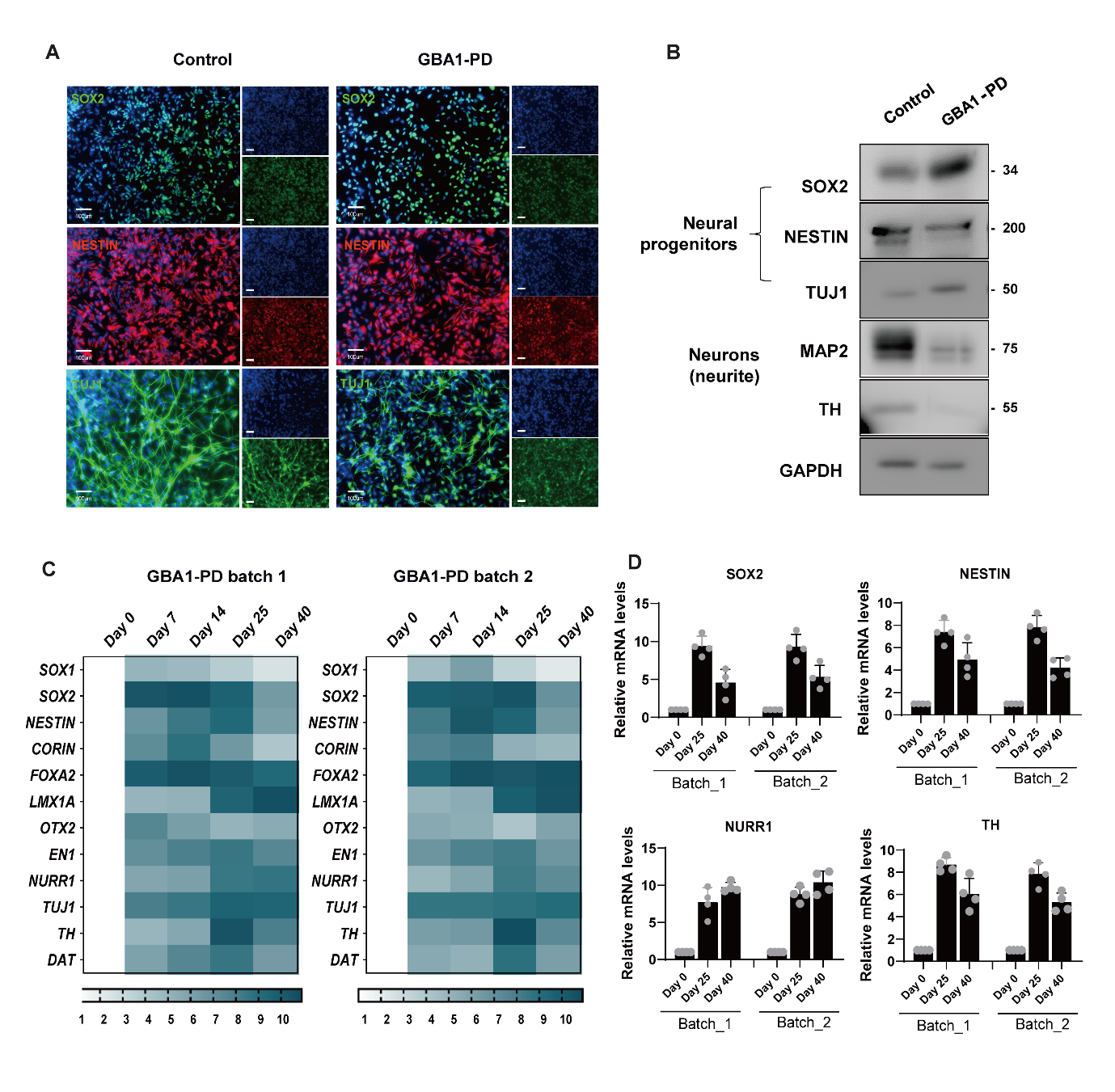
**

**Supplementary Fig. 2. Neural differentiation of normal and *GBA1*-PD hiPSCs for 25 days.** (A) Immunofluorescence analysis of control and *GBA1*-PD neural cells. They efficiently generated to neural progenitor cells (NPCs) by day 25. Scale bar: 100 μm. (B) Protein expression analysis confirming reduced levels of neural progenitor and neuron markers, including NESTIN, TUJ1, MAP2 and TH in *GBA1*-PD neural cells compared to control. (C) Heatmap of gene expression for stage-specific neural markers in midbrain dopaminergic neurons from day 0 to day 40. (D) mRNA expression levels of neural progenitor markers (SOX2 and NESTIN) and dopaminergic neuron markers (NURR1 and TH) in *GBA1*-PD-derived neural cells. Quantification data are represented as mean ± SEM (n=4).

**Supplementary Fig. 3**


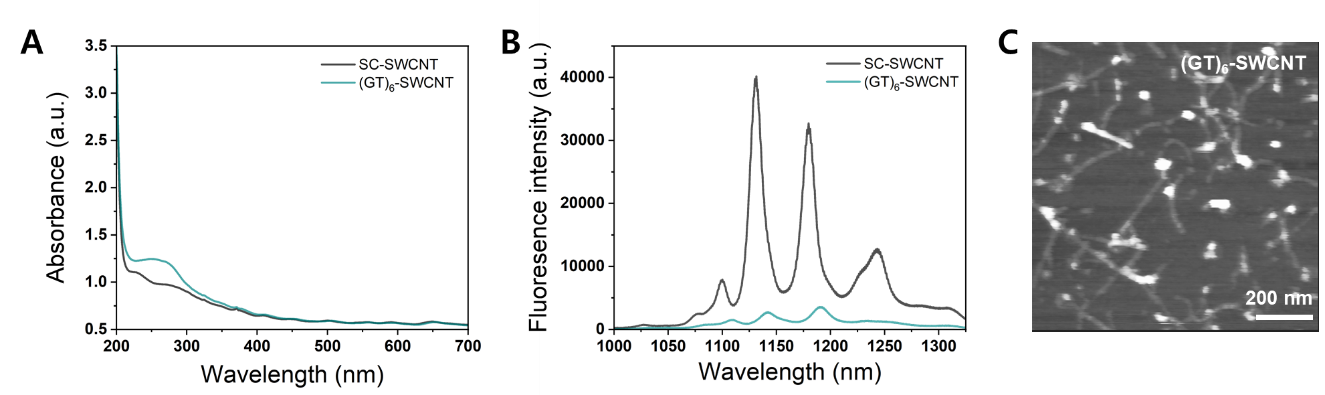


**Supplementary Fig. 3. Characterization of ssDNA-SWCNT and SC-SWCNT complexes.** The properties of ssDNA-SWCNTs were compared with those of SWCNTs dispersed using sodium cholate (SC), a widely used surfactant for noncovalent functionalization. (A) Absorption spectra of ssDNA-SWCNT and SC-SWCNT. (B) Fluorescence emission spectra of ssDNA-SWCNT and SC-SWCNT under identical SWCNT concentration conditions. (C) Atomic force microscopy (AFM) images of ssDNA-SWCNT, revealing an average length of 233 nm (lateral) and 0.9 nm (height).

**Supplementary Fig. 4**


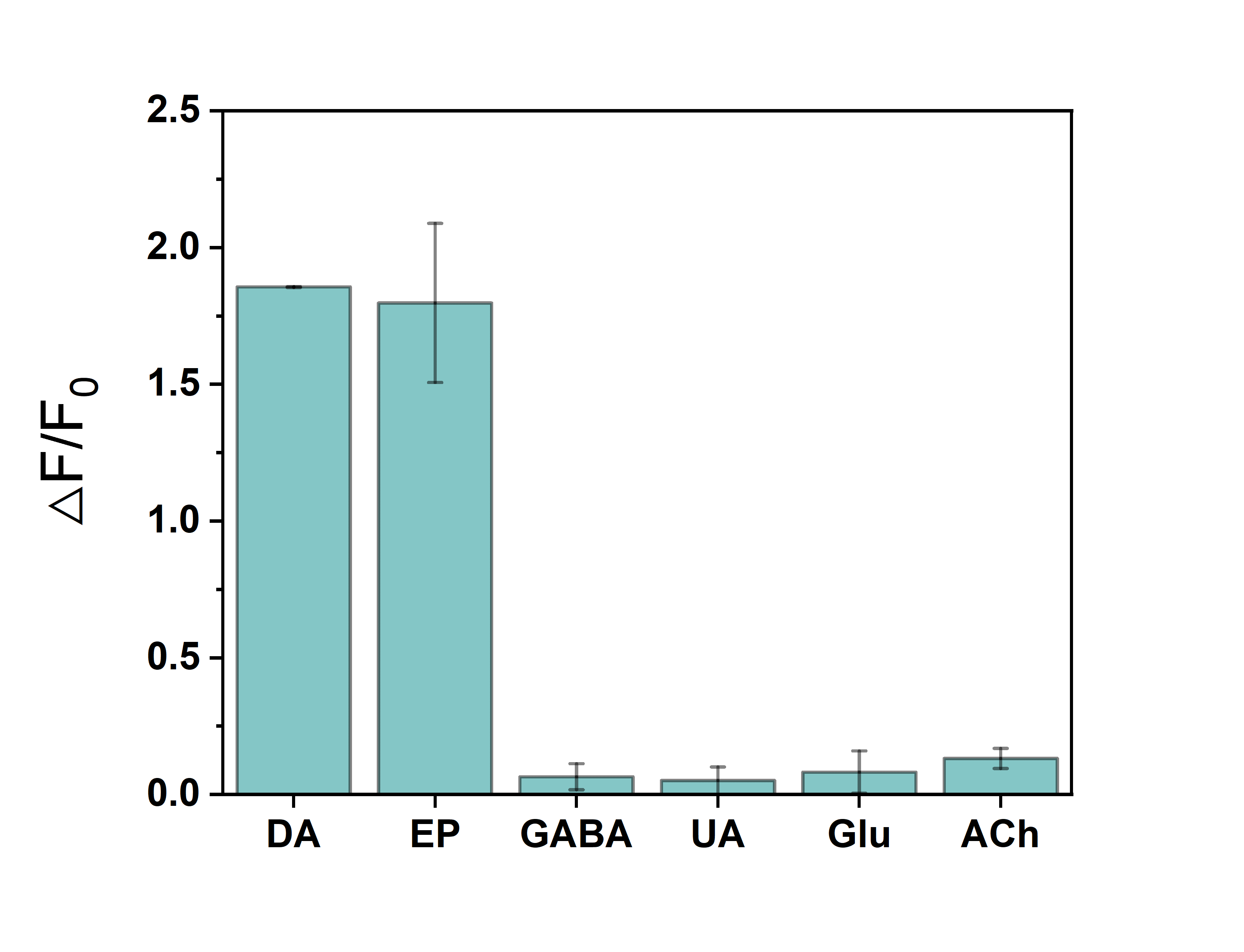


**Supplementary Fig. 4. NIRCat optical response to 100 μM DA, EP, GABA, Glu, ACh, and 10 μM uric acid (UA) in PBS solution.** The △F/F0 values were calculated at 1191 nm. Black bars represent the average of n=3 independent measurements, with error bars indicating the standard deviation (SD) of these measurements.

**Supplementary Fig. 5**

**
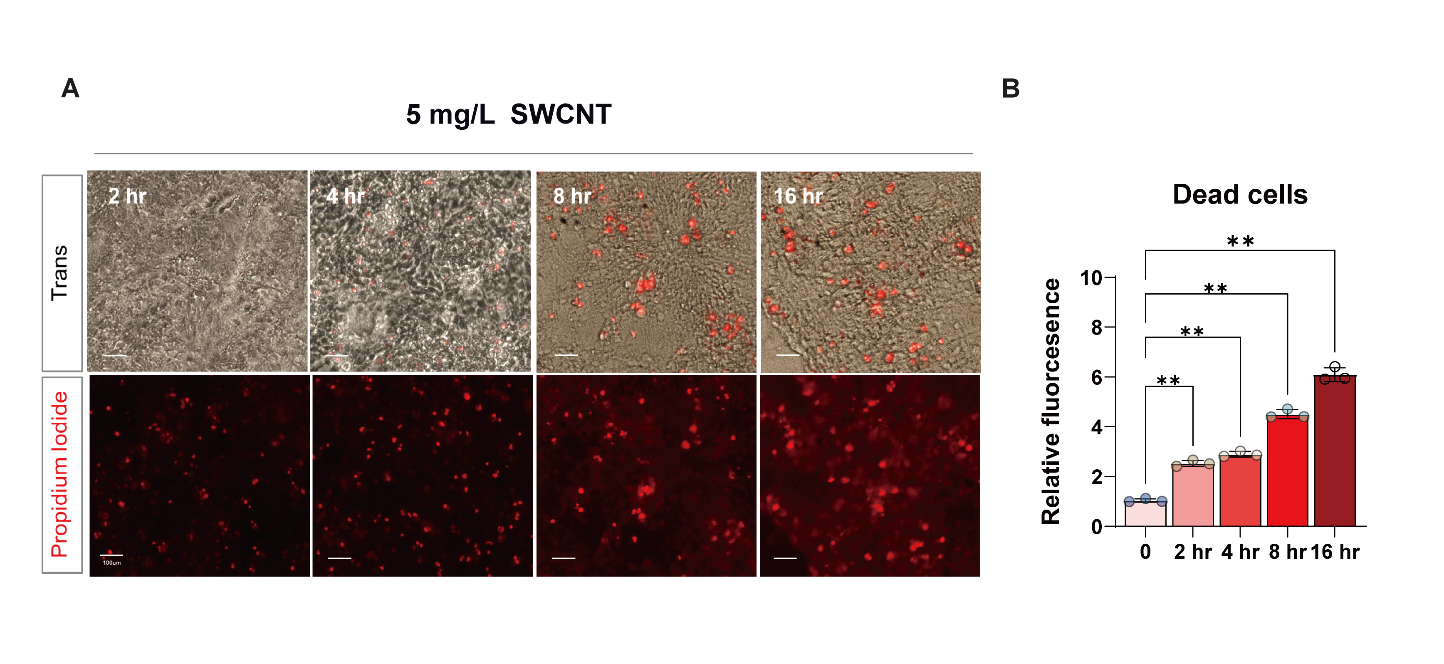
**

**Supplementary Fig. 5. PI staining for apoptotic cells exposed to 5 mg/L SWCNT from 2 h to 16h.** (A) PI staining of neural cells treated with 5 mg/L NIRCat during long term periods (2, 4, 8 and 6h), (B) Time-dependent increase in PI-positive cells, indicative of apoptosis. PI-stained cells were analyzed using ordinary one-way ANOVA, followed by Dunnett’s multiple comparison’s test: ** *p*< 0.01,

**Supplementary Fig. 6**

**
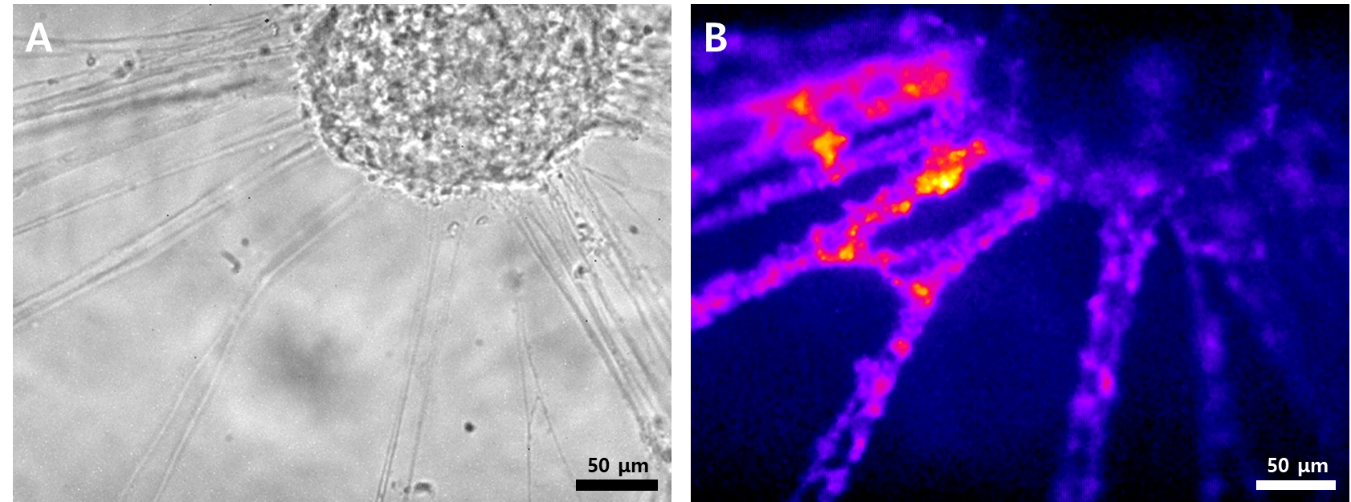
**

**Supplementary Fig. 6. Brightfield and NIR fluorescence images of cultured cells incubated with nIRCat probes on a Matrigel substrate**. (A) Brightfield image showing the morphology of the cultured cells. (B) NIR fluorescence image demonstrating the distribution of NIRCat sensors associated with the extracellular matrix and adsorbed onto the cell membrane.

**Supplementary Fig. 7**

**
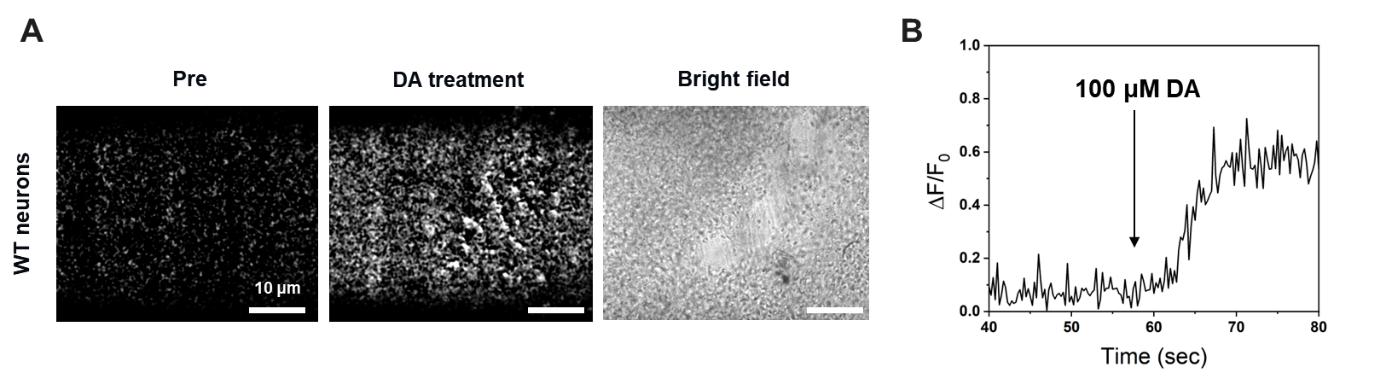
**

**Supplementary Fig. 7. NIRCat response to DA treatment** (A) Fluorescence images of NIRCat before treatment (pre) and after addition of 100 μM DA to unstimulated control neurons to confirm activation of the NIRCat nanosensors as positive control. (B) The time trace of △F/F_0_ for 100 μM DA treatment.

**Supplementary Fig. 8**

**
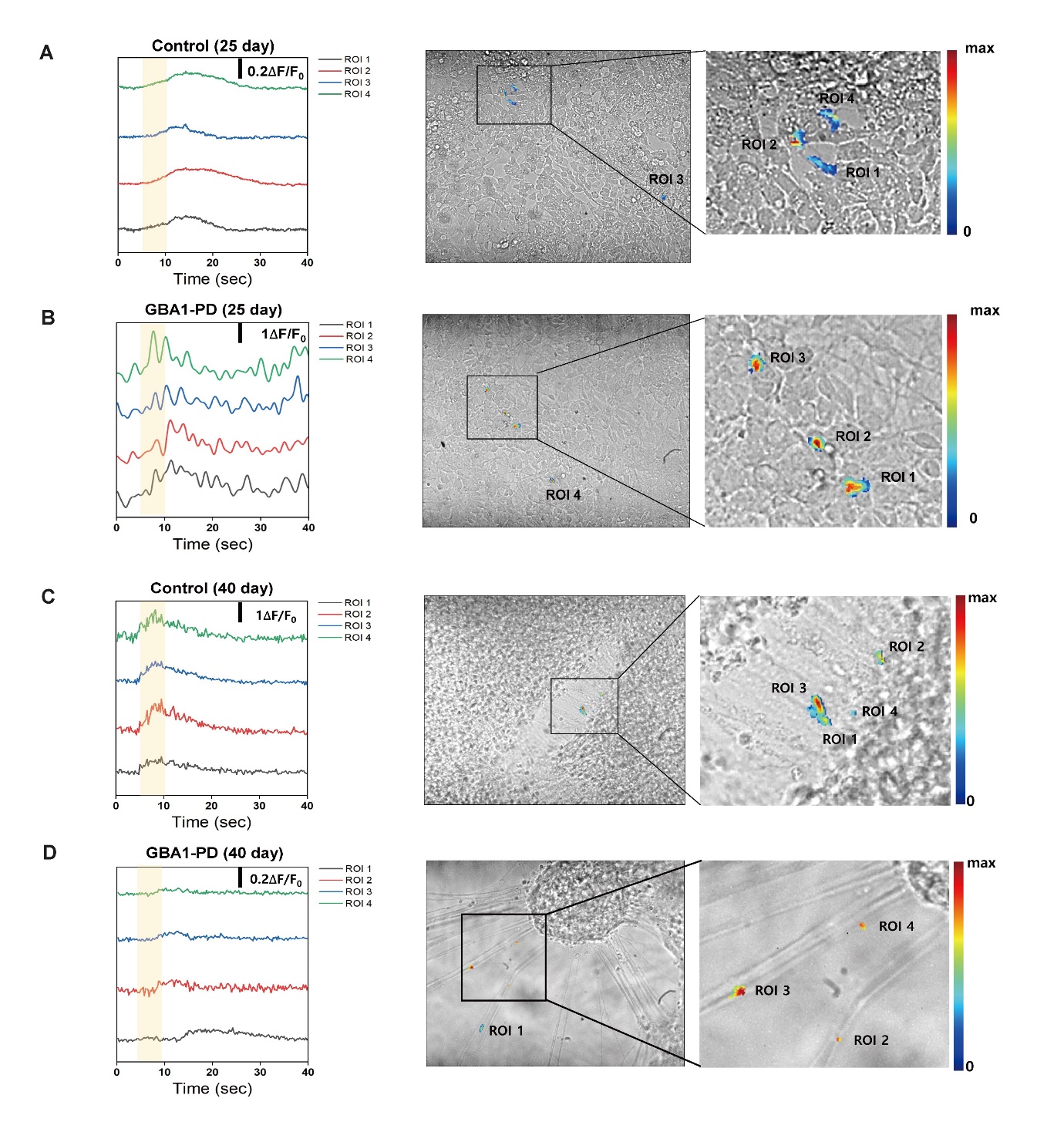
**

**Supplementary Fig. 8. Comparison of DA release in individual hotspot between control and *GBA1*-PD** (A) The time trace of △F/F0 following the 20 Hz electrical stimulation for 5 seconds (yellow box) in hotspot region of interest (ROI) in the early stage of control neurons (left). The hotspot ROIs were numbered and shown in bright field images (right). (B) The time trace of △F/F0 and its position in hotspot ROI at the early stage of *GBA1*-PD neuron. (C-D) The time trace of △F/F0 and its position in hotspot ROI at the late stage of control (C) and *GBA1*-PD neuron (D). The hotspot ROIs were observed around formed neurite.

**Supplementary Fig. 9**


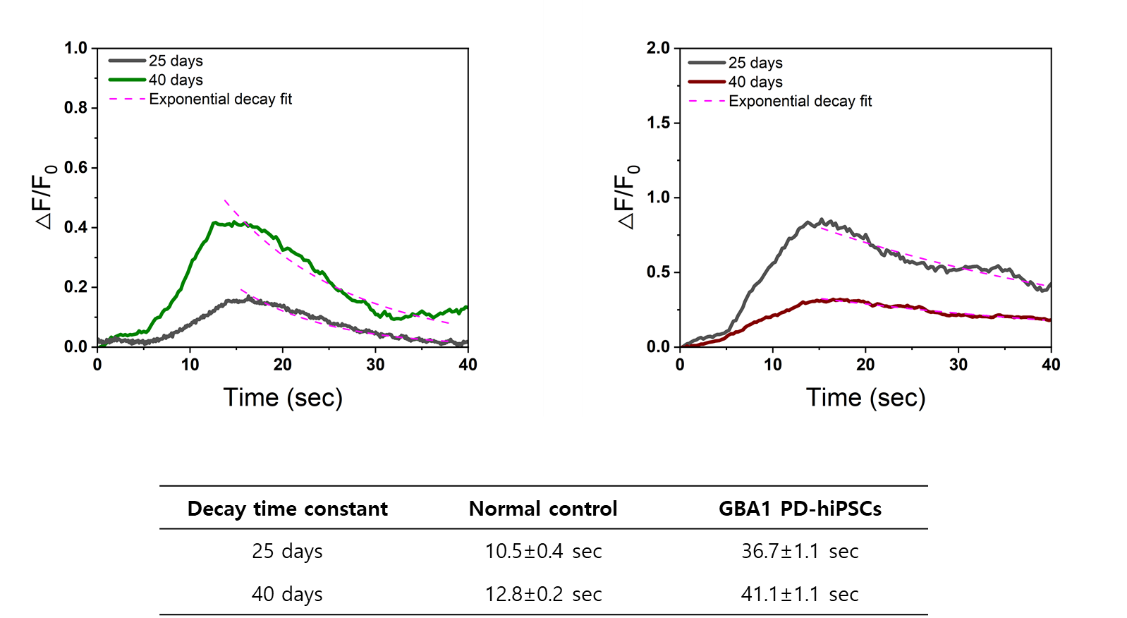


**Supplementary Fig. 9. “Turn-off” rate of NIRCat nanosensors**, which correlates dopamine clearance rates, in control and GBA1-PD neurons. Turn off rates were quantified by calculating the decay time constants following dopamine release. The decay time constant was measured using an exponential decay fitting. Data are presented as mean ± SEM.
